# Liquid biopsy as an option for predictive testing and prognosis in patients with lung cancer

**DOI:** 10.1186/s10020-021-00331-1

**Published:** 2021-07-03

**Authors:** Alvida Qvick, Bianca Stenmark, Jessica Carlsson, Johan Isaksson, Christina Karlsson, Gisela Helenius

**Affiliations:** 1 Dept. of Laboratory Medicine, Örebro University Hospital, Södra Grev. Roseng., 701 85 Örebro, Sweden; 2 Faculty of Medicine and Health, Örebro University, Örebro, Sweden; 3 Dept. of Urology, Faculty of Medicine and Health, Örebro University, Örebro, Sweden; 4 Dept. of Respiratory Medicine, Gävle Hospital, Gävle, Sweden; 5 Centre for Research and Development Region Gävleborg/Uppsala University, Gävle, Sweden; 6 Dept. of Immunology, Genetics and Pathology, Uppsala University, Uppsala, Sweden; 7 School of Health Sciences, Örebro University, Örebro, Sweden

## Abstract

**Background:**

The aim of this study was to investigate the clinical value of liquid biopsy as a primary source for variant analysis in lung cancer. In addition, we sought to characterize liquid biopsy variants and to correlate mutational load to clinical data.

**Methods:**

Circulating cell-free DNA was extracted from plasma from patients with lung cancer (n = 60) and controls with benign lung disease (n = 16). Variant analysis was performed using the AVENIO ctDNA Surveillance kit and the results were correlated to clinical and variant analysis data from tumor tissue or cytology retrieved from clinical routine diagnostics.

**Results:**

There were significantly more variants detected in lung cancer cases compared to controls (p = 0.011), but no difference between the histological subgroups of lung cancer was found (p = 0.465). Furthermore, significantly more variants were detected in patients with stage IIIb–IV disease compared to patients with stage I–IIIa (median 7 vs 4, p = 0.017). Plasma cfDNA mutational load was significantly associated with overall survival (p = 0.010). The association persisted when adjusted for stage and ECOG performance status (HR: 3.64, 95% CI 1.37–9.67, p = 0.009). Agreement between tumor and plasma samples significantly differed with stage; patients with stage IIIb–IV disease showed agreement in 88.2% of the cases with clinically relevant variants, compared to zero cases in stage I–IIIa (p = 0.004). Furthermore, one variant in EGFR, two in KRAS, and one in BRAF were detected in plasma but not in tumor samples.

**Conclusion:**

This study concludes that in the vast majority of advanced NSCLC patients a reliable variant analysis can be performed using liquid biopsy from plasma. Furthermore, we found that the number of variants in plasma is associated with prognosis, possibly indicating a strategy for closer follow up on this crucial patient group.

**Supplementary Information:**

The online version contains supplementary material available at 10.1186/s10020-021-00331-1.

## Introduction

Lately, targeted therapy and immune checkpoint inhibition have revolutionized treatment for a subset of patients with non-small cell lung cancer (NSCLC) (Tsao et al. 2016). So far, drug targets are almost exclusively found in adenocarcinomas (AC), which account for about 40% of all lung cancer cases (Tsao et al. 2016). At present, there are approved drugs for lung cancer, targeting tumor cells with mutated *EGFR*, *ALK* and *ROS1* fusions, and mutated *BRAF* (Tsao et al. 2016). Additionally, immune checkpoint inhibition that activates the immune system to target the tumor, such as monoclonal antibodies against PD1 and PD-L1, has further advanced the treatment of lung cancer (Brahmer et al. 2015).

The emergence of targeted therapies has dramatically increased the need for comprehensive tumor genotyping to predict optimal treatment as well as monitoring of the response. In turn, increased understanding of the genomic landscape of tumors has resulted in several basket trials investigating tumor agnostic treatments, with some drugs already receiving approval by the Food and Drug Administration in the US. This includes the anti-PD-1 immune checkpoint inhibitor pembrolizumab and the *NTRK* fusion-targeting larotrectinib (Patnaik et al. 2015; Le et al. 2015; Drilon et al. 2018). The indication for treatment with pembrolizumab in the agnostic setting includes a mismatch repair deficient tumor resulting in an unstable genome with a high tumor mutational burden (TMB) (https://www.fda.gov/drugs/drug-approvals-and-databases/fda-approves-pembrolizumab-adults-and-children-tmb-h-solid-tumors). However, an agnostic approach still requires tumor material, which is especially challenging in lung cancer considering the sparse tissue material obtained in many cases. Difficulties in tissue sampling is due to tumor localization and comorbidities that normally prevent surgical biopsies, and at times even core needle biopsies, and diagnosis is often based on forceps biopsies obtained by bronchoscopy or fine needle aspirates. An alternative source of tumor material could be the use of circulating biomarkers obtained for example through a blood sample.

Circulating cell-free DNA (cfDNA) is a major part of the term liquid biopsy and has been extensively studied across several cancer types with promising results (Lin et al. 2017; Gao et al. 2017; Jiang et al. 2016; Spindler et al. 2017; Zhou et al. 2016). The proportion of cfDNA that originates from the tumor, called circulating tumor DNA (ctDNA), could be used to detect diagnostic, predictive, and prognostic biomarkers (Schwarzenbach et al. 2011; Stroun et al. 1989; Nie et al. 2015; Rolfo et al. 2014). Currently, ctDNA is already in use clinically for variant detection in *EGFR* as an option for routine follow-up of resistance mutations in patients with NSCLC (Wu et al. 2018). This is based on the use of ultra-sensitive assays, such as droplet digital PCR, for specific variants. To obtain information about a larger spectrum of genetic alterations, the method of choice is currently next generation sequencing (NGS). This method has been employed in other studies to analyze *EGFR* mutations in ctDNA from patients with NSCLC (Marchetti et al. 2015; Douillard et al. 2014). However, the performance of the tests was variable, between 50 and 80% sensitivity, compared to tissue or cytology samples, and in need of further improvement for ctDNA to be established as a clinical biomarker. Sequencing of larger genomic regions, to calculate the TMB for the clinical application of response to immunotherapy, has also been performed in NSCLC on plasma (Koeppel et al. 2017; Gandara et al. 2018; Andrew et al. 2017). The studies reported some scattered results regarding the TMB agreement with tumor tissue which might indicate that the sensitivity is again at the core of the problem.

In the present study, liquid biopsy was evaluated as a primary source for clinical variant analysis using a large NGS panel of 197 genes on plasma samples from patients with lung cancer and a benign lung disease control group. Detection of targetable variants as well as agreement in variants between plasma cfDNA and tumor samples were evaluated. In addition, the total number of variants, used as a simplified version of TMB, was associated to clinical parameters.

## Materials and methods

### Study participants

Patients referred to the lung clinic at Örebro University Hospital with suspicion of lung cancer were invited to participate in the study. Samples were collected during routine investigation, prior to diagnosis. Inclusion criteria consisted of: (1) patient with suspicion of lung cancer and (2) age above 18 years. Exclusion criteria included: (1) cancer of other origin than lung, (2) sample not collected prior to diagnosis, (3) inadequate tumor material for diagnosis and (4) insufficient cfDNA quantity for analysis. This resulted in a final cohort of 61 study participants with lung cancer and 16 with benign lung diseases including different types of inflammation, fibrosis and noduli, in and around the lung tissue. The latter group was consequently used as a control group, and is hereafter referred to as such. Survival status was retrieved from medical journals (followed until 5th of February 2020) and at time of retrieval 33 of 60 cancer patients were deceased with a median follow up time of 11.0 months (Additional file 1: Table S1). The study was approved by the regional ethics committee board in Uppsala (Dnr 2015-400) and participants gave written informed consent before inclusion.

### Sample collection and extraction

Blood was collected in Cell-Free DNA™ BCT tubes (Streck, Omaha, NE) and plasma was retrieved by a two-step centrifugation; 2000×*g* for 10 min followed by 16,000×*g* for 10 min. Plasma was stored at − 80 °C until extraction of cfDNA was performed on 4 mL plasma, using the QIAsymphony DSP Circulating DNA kit on the QIAsymphony SP system (Qiagen, Germany) according to the manufacturer’s instructions.

Paired tumor samples used in clinical diagnosis, formalin-fixed and paraffin-embedded (FFPE) tissue blocks or cytological specimens, was used for comparison and are hereafter referred to as tumor samples. Tumors were staged and histologically classified according to the guidelines of the International Association for the Study of Lung Cancer and World Health Organization nomenclature, respectively (Detterbeck et al. 2017; Travis et al. 2004). DNA was extracted using FFPE DNA Purification Kit on the MagLEAD 12gc (Precision System Science, Germany) or with QIAamp DNA FFPE Tissue kit on the Qiacube (Qiagen). RNA was extracted using FFPE Total RNA Purification Kit (Exscalebio, Sweden). Concentration of extracted DNA from plasma, tissue or cytology was measured using Qubit dsDNA HS assay kit (Thermo Fisher, USA, MA) and RNA from tissue or cytology with Qubit RNA HS assay kit (Thermo Fisher) with Qubit 2.0 Fluorometer (Thermo Fisher).

### Library preparation and sequencing

Library preparation of cfDNA samples was performed with the AVENIO ctDNA Surveillance kit (Roche Diagnostics, Rotkreuz, Switzerland). Libraries were generated from 9.5 to 50 ng of cfDNA according to the protocol provided by the manufacturer. Concentration and fragment length was measured with dsDNA HS assay kit (Thermo Fisher) on Qubit 2.0 Fluorometer (Thermo Fisher) and High sensitivity D5000 kit (Agilent, USA, CA) on 4200 TapeStation Instrument (Agilent), respectively. Generated libraries were sequenced on the Illumina NextSeq 550Dx using the High output kit v2 300-cycles (Illumina, San Diego, CA, USA) to a unique molecular depth of > 500×.

DNA and RNA from tumor samples were analyzed with Ion AmpliSeq Colon and Lung Cancer Research Panel v2 (Thermo Fisher) consisting of 92 amplicons (size range 52–138 bp) from 22 genes and the Oncomine Solid Fusion transcript kit (Thermo Fisher) including four fusion transcripts, respectively (https://www.thermofisher.com/se/en/home/clinical/diagnostic-testing/condition-disease-diagnostics/oncology-diagnostics/oncomine-solid-tumour-kits.html; Laurent-Puig et al. 2016). Library and template preparation was performed on the Ion Chef System (Thermo Fisher) and sequenced on the Ion Torrent systems, either Ion PGM or Ion GeneStudio S5 Prime. For sequencing, Ion 316/318 Chip Kit V2 BC or Ion 520/530 Chip Kit was used for PGM and S5 Prime, respectively.

When tumor samples were insufficient for variant analysis with NGS, data was retrieved from clinical data repositories consisting of qPCR and Fluorescent in situ hybridization (FISH). Where tumor samples were insufficient for analysis with both DNA and RNA NGS, DNA NGS was prioritized. The collective data from variant analysis on tumor samples is further referred to as clinical variant analysis data.

A subset of tumor samples (n = 24), where material was still available, was analyzed with the AVENIO FFPE Surveillance kit (Roche Diagnostics) according to protocol, with the concentration and fragment length measured as for the cfDNA libraries.

### Mapping and variant calling

For cfDNA and tumor samples run with AVENIO assays, variants were called using the AVENIO Oncology Analysis software (version 2.0.0, Roche Diagnostics) using default settings, based on references (Newman et al. 2014, 2016; Talevich et al. 2016).

The Torrent Mapping Alignment Program (TMAP) within the Torrent Suite software version 5.10 (Thermo Fisher) was used to map sequences against the human reference genome (hg19). Default reporting of mutations was used with the addition of allowing for multiple nucleotide variant, multiple nucleotide polymorphism, and complex variants. Mutations were annotated with the Oncomine variant annotation (Thermo Fisher) within the Ion Reporter software v5.10 (Thermo Fisher). The lowest allowed depth of coverage was set to 1200× and variants were only considered if the allele frequency (AF) was > 5% in a tumor sample, with the exception of known hotspot genes with a cutoff of 3%. If a hotspot variant appeared below cutoff, NGS was rerun with a new tissue or cytology section and included in the analyses if concordant.

For clinical interpretation of cfDNA and tumor samples run with AVENIO assays, NAVIFY^®^ Mutation Profiler (version 1.1.1, Roche diagnostics) was used, which integrates data from Catalogue of Somatic Mutations in Cancer (COSMIC), The Cancer Genome Atlas (TCGA), ClinVar and Exome Aggregation Consortium (ExAC) for classification (Bamford et al. 2004; Yaung et al. 2020). For tumor samples run with Ion Ampliseq or Oncomine Solid fusion kit, the variant caller file was also run through NAVIFY^®^ Mutation Profiler for verification of consistency and additional interpretation of variants. Variants present in ExAC with a frequency > 0.01 were filtered out. Clinically relevant variants were defined in databases and lung cancer studies (Hagemann et al. 2015; Frampton et al. 2015; Kobayashi et al. 2015; https://www.mycancergenome.org/; Chakravarty et al. 2017) and included (variants targetable for treatment are marked with an asterisk): *BRAF* codon 600*; *EGFR* exon 19 deletions*, exon 20 insertions, T790M*, codon 719*, exon 18 deletion E709-T710delinsD, A763_Y764insFQEA*, S768I* (exon 20), exon 19 insertions, C797S (exon 20), L858R*, codon 851 and 861* (exon 21); *ERBB2* exon 20 insertions; *KRAS* codon 12, 13 and 61; *NRAS* codon 12, 13 and 61; *PIK3CA* codon 542, 545 and 1047 and in *MET* exon 14 splice variants* and deletions*, Y1003*, D1010*; as well as fusions with ALK*, RET* and ROS1*. All KRAS variants in codon 12 and 13 were considered clinically relevant in accordance with Swedish guidelines, albeit variable prognostic value.

### Statistics

Linear regression was used for association between sequencing performance and DNA input. Mutational load was defined as the total number of variants detected in plasma for each patient. Mann–Whitney U-test’s and Kruskal–Wallis test’s was used for analyzing differences regarding mutational load and AF between two and several groups, respectively.

Variant agreement between cfDNA and tumor samples was evaluated using Fisher’s exact test, with tumor samples as reference. Variants detected by at least one of the variant analysis methods employed, with an AF ≤ 0.01 in ExAC and classified as pathogenic in COSMIC, were considered. Tumor samples with at least one concordant variant in cfDNA were considered concordant.

The impact of the number of variants (0–3 vs 4–9 vs 10–22) on overall survival was estimated using Log-rank test and Cox proportional hazards, both by univariate and multivariate analysis. Covariates included stage (I–IIIa vs IIIb–IV) and Eastern cooperative oncology group performance status (ECOG PS; 1, 2, 3–4). All variables fulfilled the assumption of proportional hazards. Survival analysis was visualized using Kaplan Meier survival estimate.

P-values < 0.05 were considered statistically significant. Statistical analyses were performed using IBM SPSS statistics version 25.0 (SPSS Inc. Chicago, IL) and R version 4.0.2 with packages ggplot2 version 3.3.2 and GenVisR version 1.20.0 (Wickham 2016; Skidmore et al. 2016).

## Results

### Cohort characteristics

The cohort consisted of 77 study participants attending the lung clinic for investigation of lung cancer where a plasma sample was available for cfDNA variant analysis with NGS (Table 1, Additional file 1: Table S1). One cfDNA plasma sample was excluded due to < 500× unique molecular depth (UMD). Participants were diagnosed with NSCLC (AC; n = 37), NSCLC squamous cell carcinoma (SqCC; n = 15), SCLC (n = 8) or benign lung disease (controls; n = 16).

**Table 1:** Cohort characteristics

Characteristic	Lung cancer n (%) (n = 60 ^b^)	Controls n (%) (n = 16)^d^	Lung cancer vs controls, p-value	Total N (%) (N = 76)
Age			0.126	
Median	72	75		72
Range	39–85	49–84		39–85
Sex			0.835	
Male	32 (53.3)	9 (56.3)		40 (52.6)
Female	28 (46.7)	7 (43.8)		36 (47.4)
Smoking			0.391	
Never	12 (20.0)	6 (37.5)		19 (25.0)
Former	29 (48.3)	6 (37.5)		34 (44.7)
Current	19 (31.7)	4 (25.0)		23 (30.3)
Histology				
AC	37 (61.7)			37 (48.7)
SqCC	15 (25.0)			15 (19.7)
SCLC	8 (13.3)			8 (10.5)
Controls				16 (21.1)
Stage				
I–IIIa	19 (31.7)			
IIIb–IV	41^c^ (68.3)			
ECOG PS				
PS 0	15 (25.0)			
PS 1	24 (40.0)			
PS 2	12 (20.0)			
PS 3–4^a^	9 (15.0)			
Treatment				
Surgery	9 (15.0)			
Radiation	9 (15.0)			
Chemotherapy	23 (38.3)			
Targeted therapy or immunotherapy	10 (16.7)			
Best supportive care/no treatment	9 (15.0)			

Controls consisted of patients referred to the lung clinic with the suspicion of lung cancer but later diagnosed with different benign lung diseases

*AC* adenocarcinoma, *SqCC* squamous cell carcinoma, *SCLC* small cell lung cancer, *ECOG PS* Eastern cooperative oncology group performance status

^a^One patient had PS 4

^b^One cfDNA sample was excluded due to low unique molecular depth

^c^Five patients had stage IIIb

^d^Controls with benign lung disease were diagnosed with different types of inflammation, fibrosis and noduli in and around the lung tissue

The median age for lung cancer patients (n = 60) was 72 years, with a slightly higher proportion of males (53.3%, n = 32) and former smokers (48.3%, n = 29) compared to never (20.0%, n = 12) and current smokers (31.7%, n = 19). Stage IIIb–IV disease was observed in 60.7% (n = 41) of cases, and the most common first line treatment was chemotherapy (38.3%, n = 23).

Control patients with benign lung disease (n = 16) had a median age of 75 years, with 56.3% (n = 9) being males. Smoking status was evenly distributed between never and former smokers (37.5%, n = 6), with less current smokers (25.0%, n = 4).

### Variant agreement between plasma and tumor samples

**Fig. 1:**
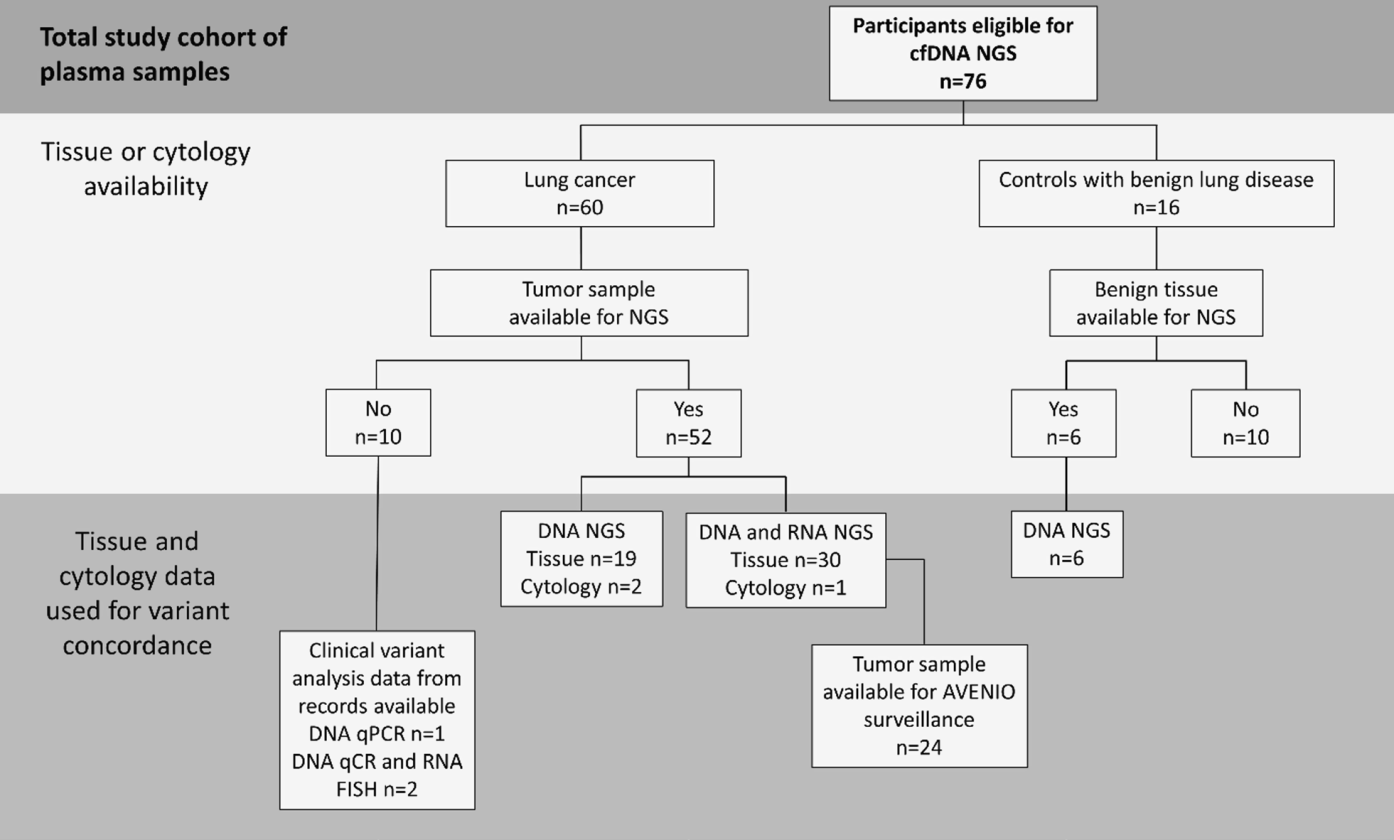
Inclusion for variant agreement between tumor and plasma samples. The total study cohort consisted of patients with cfDNA AVENIO NGS performed (n = 77). One cfDNA plasma sample was excluded due to too low unique molecular depth (< 500×). For agreement between tumor or benign lung tissue and paired plasma, clinical variant analysis data from DNA and RNA NGS was retrieved. When tumor tissue or cytology was insufficient for NGS, clinical variant analysis data was retrieved from qPCR or FISH for DNA or RNA, respectively. A subset of patients (n = 24), where additional tumor material was available, was also analyzed with the corresponding AVENIO kit as for plasma

This study used real-world clinical data and patients were included based on the availability of a cfDNA plasma sample, regardless if the quality or quantity of the tissue or cytology sample was sufficient for variant analysis. However, for agreement, a variant had to be detected in a tumor sample in order to be included. Tumor sample NGS data was available for DNA and RNA variant analysis in 86.7% (n = 52) and 52.5% (n = 31) of the cases, respectively. When tumor material was insufficient for NGS, DNA and RNA variant analysis was performed with qPCR (4.9%, n = 3) and FISH (27.9%, n = 17), respectively. Collectively, this is referred to as clinical variant analysis data. Tumor sample was insufficient for variant analysis in 8.3% (n = 5) of the cases. Twenty-four samples had tumor sample available for further analysis with the AVENIO surveillance panel, where five tumor samples were excluded due to low exon coverage uniformity (< 40% of exons had > 300× coverage). In the control group, six patients had tissue available for DNA variant analysis (37.5%). A flow chart of methods used for variant analysis in tumor samples in the study cohort is shown in Fig. 1.

#### Agreement in all analyzed variants

For tumor samples with clinical variant analysis data available, the number of variants ranged from 1 to 2 (Additional file 2: Full VCF data). Across histologies, variants were detected in 22 ACs, 7 SqCCs and 1 of the SCLC tumor samples. For the 19 samples analyzed with AVENIO surveillance panel there was an addition, to the samples already positive in clinical variant analysis, of 3 ACs and 2 SqCCs with variants detected. Mean UMD of tumor samples analyzed with AVENIO surveillance panel was 1589× (n = 19, range 309–3261×) and the number of detected variants ranged from 1 to 12 (median 4). In total, 35 patients had variants detected in tumor samples and were consequently eligible for agreement analysis. The overall variant agreement of tumor and plasma samples was 62.9% (22/35), where 54.3% (19/35) had a complete agreement in all variants detected.

The AF of concordant and discordant variants in tumor samples were highly overlapping and ranged from 3.2 to 68% (median 18%) for concordant variants, and from 5.5 to 68% (median 20%) for discordant variants, respectively. Tumor sample variants that were concordant in cfDNA ranged from 0.07 to 16.93% (median 1.9%) in AF. The concordant variant with the lowest AF in plasma was 0.07% in the *BRAF* gene.

Variant agreement significantly differed with stage, where patients with stage IIIb–IV disease were 10.8 times more likely to display a concordant result compared to stage I–IIIa (stage I–IIIa, 25.0%, 3/12; and stage IIIb–IV, 82.9%, 19/23; p = 0.002). There was no difference in agreement between AC and SqCC (15/25 and 7/9, p = 0.23). Several mutations below the detection threshold of 5% for tumor samples were also confirmed in cfDNA (data not shown). In the control group, 6 patients had tissue available for mutational analysis where one of the patients had two mutations observed in tissue that were not concordant with cfDNA.

#### Agreement in clinically relevant variants

For assessment of variant agreement between plasma and tumor samples in clinically relevant genes; hotspot variants in *EGFR* (7), *KRAS* (11), and *PIK3CA* (1)*,* and fusions in *ALK* (3) and *ROS1* (1) were detected in tumor samples. All had one clinically relevant variant and agreement was therefore evaluated in a binary mode (Table 2).

**Table 2:** Variant agreement between tumor and plasma samples in variants detected in clinically relevant genes

Study ID	Gene	Variant	Tumor cell content (%)	Input cfDNA (ng)	AF (%)	
					Tumor samples	cfDNA
Concordant variants						
4	KRAS	p.Gly12Cys	50	37.3	52.85	3.06
5	KRAS	p.Gly12Val^b^	80	27.0	NA	2.09
9	EGFR	p.Glu746_Ala750del	25	11.9	45.35	5.11
18	KRAS	p.Gly12Cys	30	50.0	26.93	1.02
25	EGFR	p.Leu747_Pro753delinsSer	20	50.0	45.83	2.28
29	PIK3CA	p.Glu545Lys	20	44.7	20.41	8.10
35	ALK;EML4	Fusion	10	20.0	NA	NA
42	KRAS	p.Gly12Cys	70	47.0	46.65	5.92
51	KRAS	p.Gly13Asp	30	33.4	15.38	0.13
52	KRAS	p.Gly13Asp	30	27.2	31.96	3.22
55	EGFR	p.Leu747_Glu749del	20	20.5	39.53	5.80
67	KRAS	p.Gly12Cys	10	50.0	19.01	3.38
70	ALK;EML4	Fusion^c^	10	13.9	NA	NA
72	EGFR	p.Leu858Arg	NA^a^	50.0	1.65	0.19
75	KRAS	p.Gly12Cys	< 10	43.9	11.05	0.52
Discordant variants						
1	EGFR	p.Glu746_Ala750del^b^	60	19.8	NA	–
7	KRAS	p.Gly12Ala	70	15.6	12.43	–
21	EGFR	p.Glu746_Ser752delinsVal	20	22.7	27.71	–
32	ROS1	Fusion^c^	80	25.8	NA	–
33	EGFR	p.Glu746_Ala750del	30	29.1	62.85	–
43	KRAS	p.Gly12Cys	5	35.7	8.15	–
60	ALK;EML4	Fusion	25	22.5	NA	–
73	KRAS	p.Gly12Ala	20	38.1	15.08	–

All variants detected in clinically relevant genes in tumor samples are included and each line represents one case

*AF* allele frequency

^a^Cytological sample

^b^qPCR data from clinical records

^c^FISH data from clinical records

The overall agreement between tumor and plasma samples for clinically relevant variants was 65.2% (15/23). As with the agreement considering all variants, a concordant result in clinically relevant genes was significantly more common with stage IIIb–IV disease, where neither of the patients with stage I–IIIa (0/6) had a concordant result, compared to 88.2%, of patients with a stage IIIb–IV disease (15/17, p < 0.001). One patient had a targetable *EGFR* variant detected in plasma that was not detected in the tumor sample. Plasma detected and tumor sample absent variants were also observed in two other patients, harboring one *KRAS* and one *BRAF* mutation, respectively.

A total of 7 lung cancer patients had insufficient tumor material for mutational analysis, where one patient had a detectable *KRAS* mutation in cfDNA. Tumor material was also unavailable in 10 of the controls, with two having detectable variants in *KRAS,* of which one was a clinically relevant variant (p.Gly12Ala, AF: 0.06%). Four tumor samples with confirmed fusions from clinical variant analysis, three in *ALK* and one in *ROS1*, were further analyzed with AVENIO FFPE, where neither of the fusions were detected. In contrast, two of the four fusions were detected in cfDNA.

### Variant characteristics in plasma

The entire cohort of plasma samples (n = 76) was analyzed using the AVENIO surveillance panel and variant characteristics in plasma was further explored distinct from tumor sample data. The mean UMD of plasma samples was 4871× (n = 76, range 713×–9243×). Input of cfDNA was associated with UMD (p < 0.001), but not with overall number of detected variants (p = 0.878), also referred to as mutational load.

**Fig. 2:**
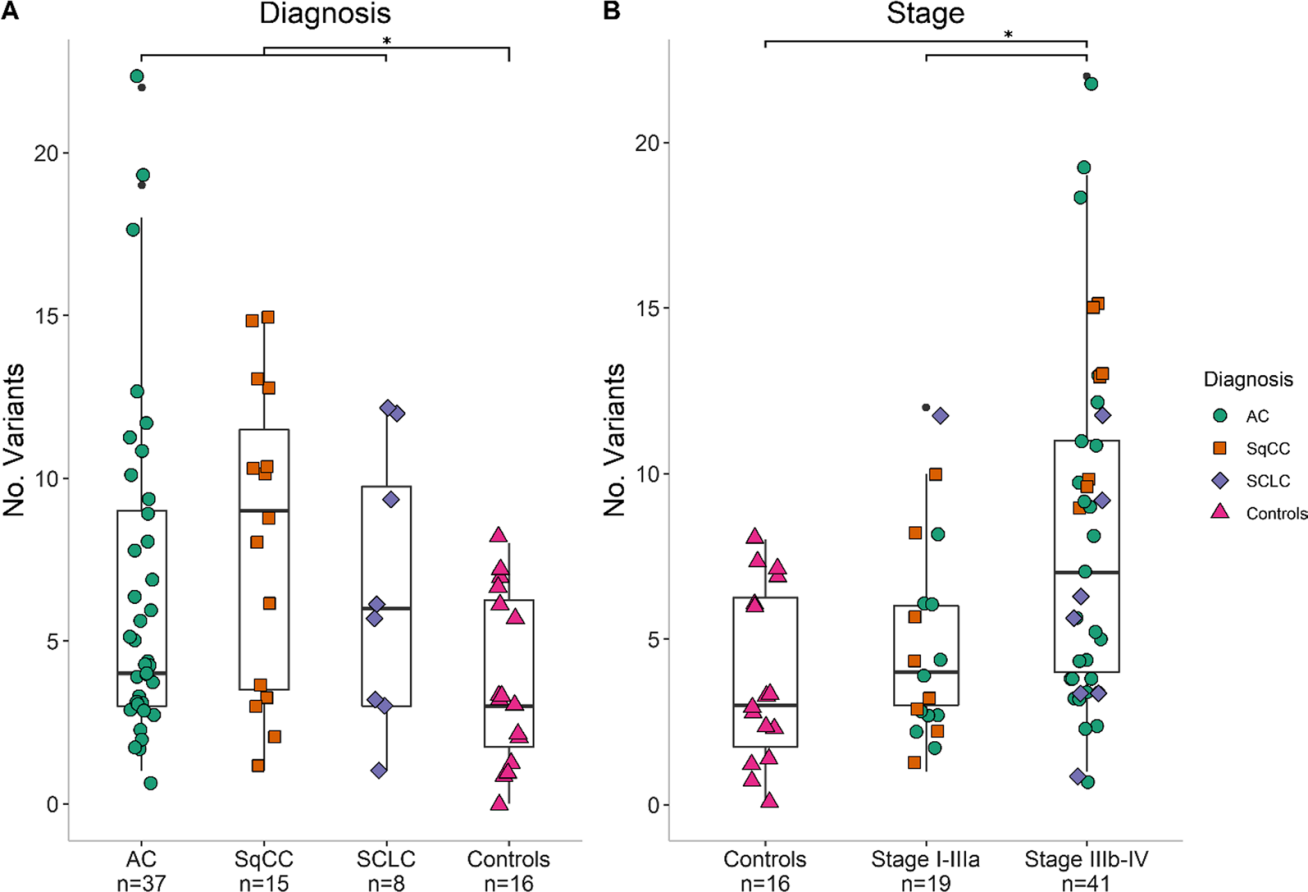
Number of variants detected in plasma cfDNA. **A** Number of variants in plasma separated on diagnosis. Controls consisted of patients referred to the lung clinic with the suspicion of lung cancer but later diagnosed with different benign lung diseases. **B** Number of variants in plasma separated on stage. *AC* adenocarcinoma, *SqCC* squamous cell carcinoma, *SCLC* small cell lung cancer

The median number of variants detected in cfDNA was 5 for AC, 9 for SqCC, 6 for SCLC and 3 for controls with benign lung disease (Fig. 2A, Additional file 2: Full VCF data). There were significantly fewer variants detected in control patients compared to patients with lung cancer (median 3 vs 6, p = 0.011), but there was no significant difference between the different histologies of lung cancer (p = 0.465). There was a significantly higher mutational load in patients with stage IIIb–IV disease compared to patients with stage I–IIIa disease (median 7 vs 4 variants, p = 0.017; Fig. 2B) and to controls (median 7 vs 3 variants, p = 0.003; Fig. 2B). There was a difference in mutational load regarding smoking status between never smokers and current/former smokers (4 variants vs 6 variants, p = 0.028). In a logistic regression, accounting for smoking status, an increase in mutational load resulted in a 24% higher odds of having a stage IIIb–IV tumor (p = 0.012, 95% CI 1.05–1.47).

AF of variants detected in plasma cfDNA ranged from 0.03 to 19.44% (median 0.25%). There was no association between AF and smoking status, (0.26%, never vs 0.32%, former/current, p = 0.237). There was no difference in AF between lung cancer and controls (p = 0.537) or between different histologies (p = 0.838) and stage (p = 0.287). Some cfDNA samples had several single nucleotide polymorphisms present at low AF. This was more common in SCLC where three samples had 50% of the low AF variants present in EXAC at high frequencies.

**Fig. 3:**
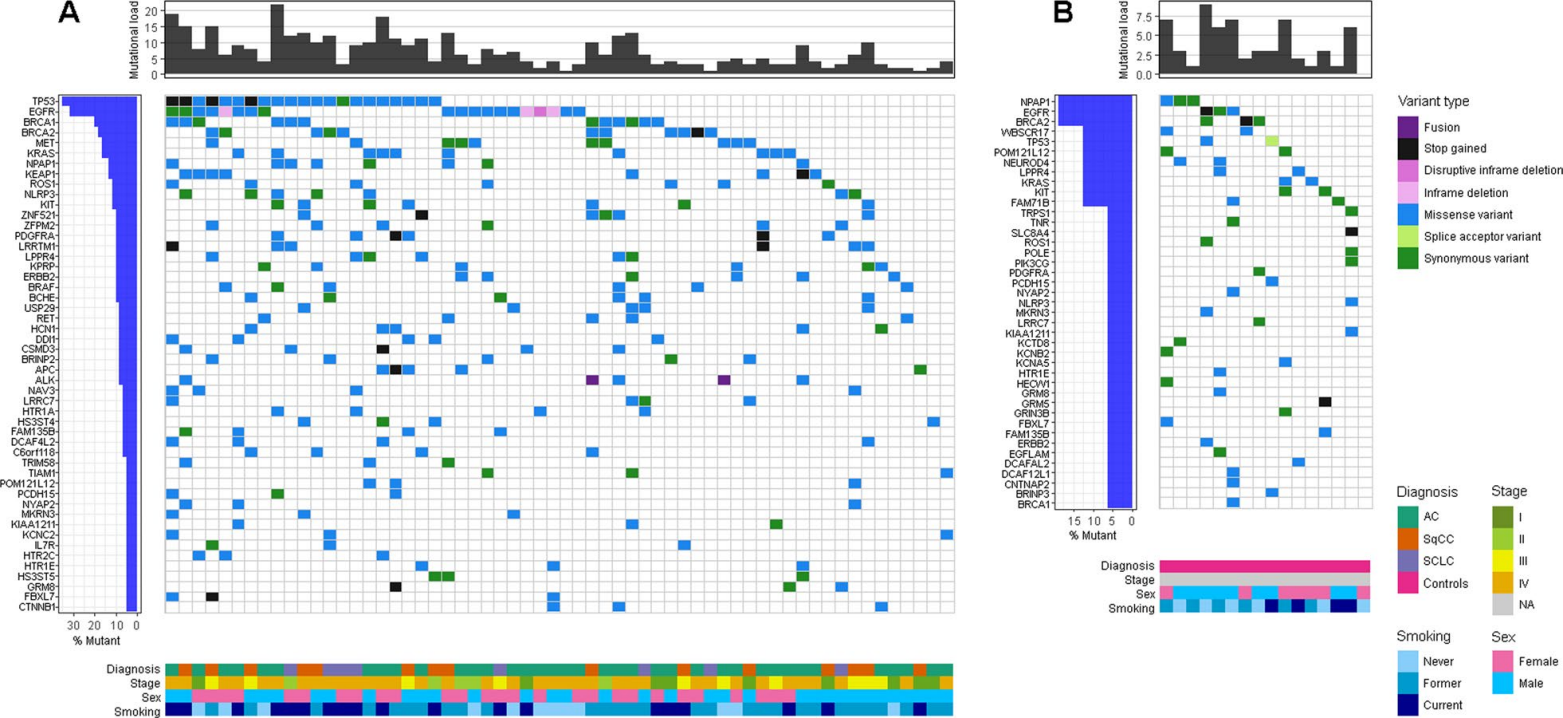
Variant characteristics in plasma. **A** Variants in the lung cancer cohort. Top 50 most commonly mutated genes are shown. **B** Variants in the control group. All genes with detected variants are shown. Controls consisted of patients referred to the lung clinic with the suspicion of lung cancer but later diagnosed with different benign lung diseases. *AC* adenocarcinoma, *SqCC* squamous cell carcinoma, *SCLC* small cell lung cancer

The most commonly mutated genes in the total lung cancer patient cohort were *TP53* and *EGFR*, with variants in 36.7% (22/60) of participants each (Fig. 3A). When analyzing different histologies separately, *TP53* remained the most mutated gene in both SqCC (46.7%, 7/15) and SCLC (50.0%, 4/8), but the second most common in AC (29.7%, 11/37). The second most common genes with variants in SqCC were *KIT* and *EGFR* in 33.3% (5/15) of cases each, where neither of the detected *EGFR* variants were associated with response to EGFR tyrosine kinase inhibitors. For AC, *EGFR* was the most commonly mutated gene present in 37.8% (14/37) of cases, out of which 35.7% (5/14) of cases were targetable. The third most common gene with variants was *KRAS* (24.3%, 9/37) and the fourth was shared by *ERBB2* and *MET* (18.9%, 7/37). The SCLC group was too small to examine further. In the control group, *BRCA2* and *NPAP1* were the most commonly mutated genes present in 18.8% (3/16) of cases each (Fig. 3B). Variants in both *BRCA1* and *BRCA2* were present across all histologies of lung cancer and controls, with a prevalence of 20.0% (12/37) and 6.3% (1/16) for *BRCA1*, and 18.3% (11/37) and 18.8% (3/16) for *BRCA2* in lung cancer patients and controls, respectively. The remaining variants were distributed at low frequencies across numerous genes. The AVENIO software detects copy number variants (CNVs) in *EGFR*, *MET* and *ERBB2* genes. In total, four positive CNVs were detected. Two patients, one with AC and one with SCLC, had CNVs in *EGFR* while a third patient with SCLC had CNVs in both *EGFR* and *MET*; corresponding to 25% (2/8) and 2.6% (1/37) CNV positivity in SCLC and AC subgroups, respectively.

**Fig. 4:**
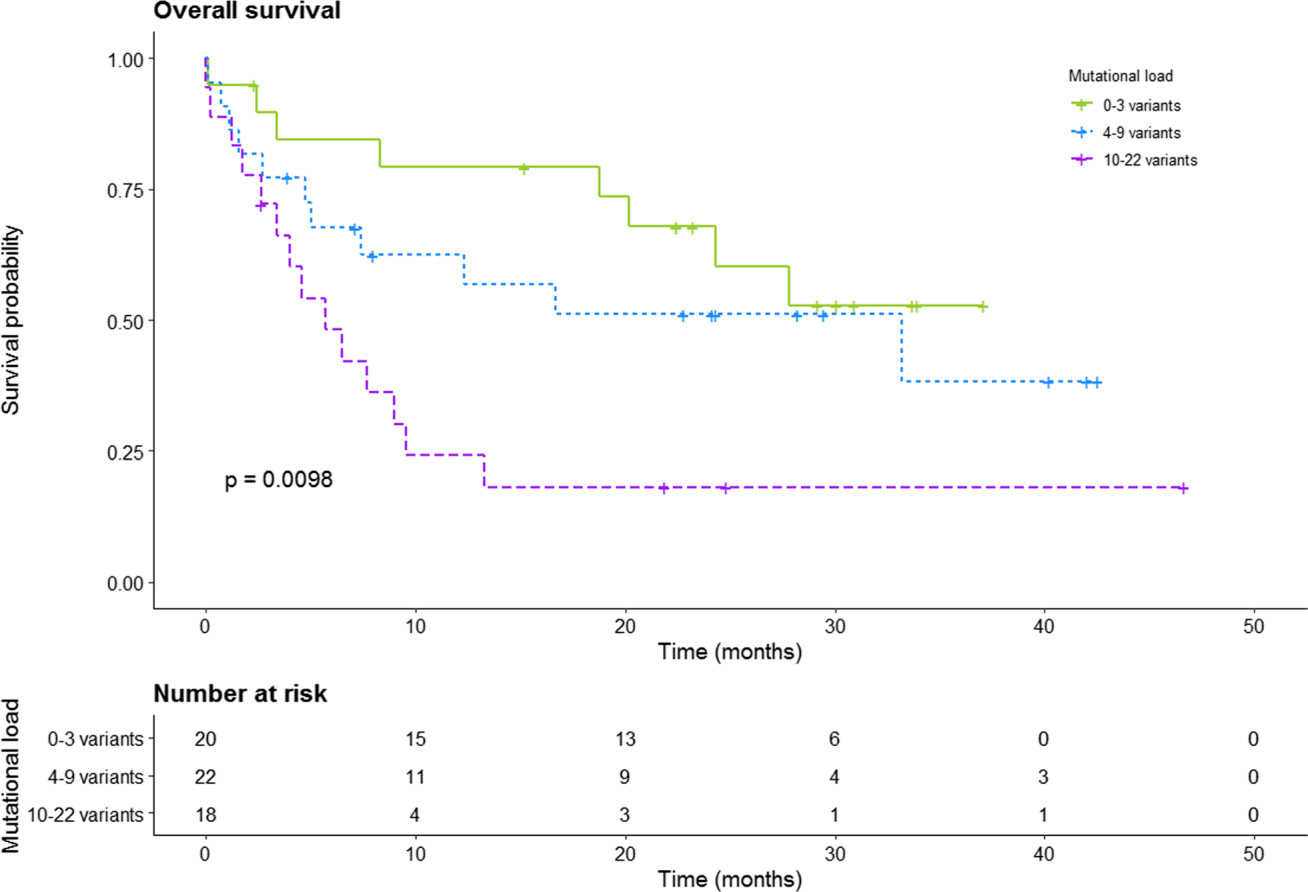
Overall survival rate of lung cancer patients stratified by number of variants in plasma. Patients were divided into groups based on number of variants 0–3 variants (n = 20), 4–9 variants (n = 23), and 10–22 variants (n = 18)

For survival analysis, patients with lung cancer were divided into three groups based on mutational load in cfDNA: 0–3 variants (n = 20); 4–9 variants (n = 22); and ≥ 10 variants (n = 18). In a univariate model, cfDNA mutational load was significantly associated with overall survival (p = 0.0098, log rank test; Fig. 4). This association persisted between patients with 0–3 and 10–22 variants in a multivariate model adjusted for stage and ECOG PS (HR: 3.64, 95% CI 1.37–9.67, p = 0.009; Table 3).

**Table 3:** Cox regression models of mutational load on survival

Variable	Univariate model			Multivariate model		
	HR	95% CI	p-value	HR	95% CI	p-value
Mutational load						
0–3 variants	1 (ref)			1 (ref)		
4–9 variants	1.58	0.63–3.93	0.328	1.95	0.72–5.32	0.192
10–22 variants	3.51	1.45–8.52	0.006	3.64	1.37–9.67	0.009
Stage						
I–IIIa				1 (ref)		
IIIb–IV				1.898	0.70–5.11	0.205
ECOG PS						
PS 0				1 (ref)		
PS 1				1.19	0.39–3.70	0.76
PS 2				3.30	1.06–10.31	0.04
PS 3–4				76.05	14.95–386.85	0.000

*ECOG PS* Eastern cooperative oncology group performance status, *HR* hazard ratio, *CI* confidence interval

## Discussion

In the present study, we investigated the use of cfDNA in plasma as a primary source for molecular variant diagnostics in a cohort of patients with lung cancer. We found that the mutational load in cfDNA was associated with overall survival in a multivariate model. In addition, a high agreement of variants between tumor and plasma samples in patients presenting with stage IIIb–IV disease was observed, but also a number of variants in plasma that could not be confirmed in tumor samples.

The most frequently mutated gene in plasma was *TP53* with a frequency of 33.8% in the total cohort and an enrichment in SqCC and SCLC (46.7% and 50.0%, respectively), compared to AC (31.6%). This is consistent with previous findings where variants in *TP53* is more common in SqCC compared to AC (Gibbons et al. 2014). Also consistent with the literature is the presence of *EGFR* variants in 36.8% in AC, where 13.5% in total was targetable by tyrosine kinase inhibitors. However, 33.3% EGFR positivity in SqCC in our cohort is aberrantly high since this histology is, almost exclusively, *EGFR* negative (Rekhtman et al. 2012). However, neither of these variants were targetable and could be explained as possible passenger mutations.

In this study, mutational load, defined as the total number of variants detected in cfDNA, was used as a simplified version of TMB. We found that mutational load in cfDNA plasma samples was associated to prognosis. Mutational load in tissue, measured by TMB, has been associated to prognosis of lung cancer previously, but international consensus guidelines concerning choice of sequencing method and cutoff values have not been reached. TMB has not been as thoroughly investigated in cfDNA and, as previously mentioned, several studies have reported a varying agreement in TMB between tissue and cfDNA (Koeppel et al. 2017; Gandara et al. 2018; Andrew et al. 2017). Müller et al. found a high correlation of TMB results when using whole genome sequencing and targeted sequencing using the CAPP-seq panel of 134 genes, a smaller predecessor of the panel used herein (Muller et al. 2017). In our study, the input amount of cfDNA was not associated with the total number of variants detected, contrary to a previous report (Koeppel et al. 2017). Despite the scattered results of cfDNA TMB, it may still be clinically useful since TMB has shown promising results for predicting response and prognosis of lung cancer patients treated with immunotherapy (Gandara et al. 2018; Khagi et al. 2017). Regarding other cancer types, one study on breast cancer patients treated with surgery and radiotherapy has shown an association between the number of cfDNA variants and relapse-free survival (Kujala et al. 2020). Jaiswal et al. (2014) found that the presence of a somatic mutation in peripheral blood cells was associated with an increase in all-cause mortality. To our knowledge, the present study is the first to report an association between the number of variants in plasma and prognosis for patients with lung cancer, irrespective of treatment.

We found a higher variant agreement between plasma and tumor samples when analyzing stage IIIb–IV disease, compared to stage I–IIIa disease, which is consistent with previous reports (Jiang and Yao 2020; Karlovich and Sun 2016). An increased likelihood of detecting a variant in an advanced stage disease is related to its elevated ability to shed DNA into the bloodstream. Apart from stage, the shedding ability has been associated to tumor size, metastatic location and genomic subtype (Lam et al. 2020; Cho et al. 2020). This adds further information for concordant and discordant variants while, unfortunately, this cohort did not have enough sample size to evaluate this further.

At present, targeted drugs are only prescribed to patients with a IIIb–IV stage disease, and is consequently in a greater need of variant analysis compared to patients with stage I–IIIa disease (https://kunskapsbanken.cancercentrum.se/diagnoser/lungcancer/vardprogram/). Our result of 82.9% total agreement, and 88.2% agreement of clinically relevant variants, between plasma and tumor samples in stage IIIb–IV disease is given further clinical value when considering the combined fail rate of obtaining a tissue biopsy and it in turn having sufficient quality for NGS analysis. As previously mentioned, an advanced disease is highly associated with an overall decline in general health status, which also corresponds to an increased fail rate for tissue sampling. In previous studies, this fail rate has been 19–51% and considering the fraction of patients with NSCLC having a targetable variant, it is highly likely that several patients eligible for targeted therapy are wrongly omitted (Douillard et al. 2014; Aggarwal et al. 2019; Meric-Bernstam et al. 2015; Hellmann et al. 2018; Thompson and Troxel 2016). In this study, the fail rate of tissue sampling was much lower, but there was still one clinically relevant mutation in *KRAS* detected in cfDNA corresponding to one of the insufficient tumor samples. In addition, three patients with tumor sample analysis results had clinically relevant variants detected in cfDNA that were undetectable in tumor samples. One of these patients had a targetable *EGFR* variant that would possibly have benefitted from targeted therapy. Even when disregarding the clinically relevant variants only detected in plasma, our results imply that for patients with stage IIIb–IV disease, to which the currently available targeted drugs are prescribed, nine out of ten patients would have sufficed with molecular variant diagnostics on plasma instead of tissue or cytology. Since plasma is a non-invasive and easily obtained tumor material source compared to a biopsy, a simple blood draw could have spared these patients from a painful attempt to repeat a biopsy with insufficient material for variant analysis.

In our cohort, several detected variants in plasma could not be verified in tumor samples when analyzing all variants. The number of detected variants in plasma was higher in patients with lung cancer compared to the control group in this study, suggesting a tumor origin for a large part of the variants. Non-verified variants in tumor samples could be due to tumor heterogeneity or they could originate from clonal hematopoiesis (CH) or other non-tumor cells. Challenges with contribution of CH regarding variants in plasma has been reported previously where Liu et al. observed that 75% of variants detected in healthy controls older than 50 years originated from CH. On the other hand, Coombs et al. (2017) reported that 25% of the plasma variants in patients with solid tumors originated from CH (Liu et al. 2019). Out of the nine most commonly mutated genes in CH, accounting for the majority of CH contribution, the panel used in the present study only contained one, *TP53*. Contrary to previous reports, *TP53* variants were not frequently detected in the control group in the present study (Hu et al. 2018; Fernandez-Cuesta et al. 2016). However, *BRCA* genes appeared with a number of variants at low AF across several histologies, including controls, and were not classified as germline. This diverges from what has previously been reported, where to our knowledge only one case with a somatic *BRCA* variant has been described in lung cancer, in a population with a high *BRCA* variant prevalence, and any *BRCA* variant presence in CH has not been reported (Kadouri et al. 2019). A lot is still unknown about the origin of variants in plasma and this is an intriguing area for future research to better understand the biological background in plasma.

Three cases with CNVs in *EGFR* and *MET* were detected in this study. One patient presented with an amplification of both *EGFR* and *MET,* which suggests an aneuploidy event on chromosome 7 (Zojer et al. 2000). This patient, and one with an amplification in *EGFR* only, were both diagnosed with SCLC. There have been no previous reports of amplifications in these genes in SCLC. In contrast, a decreased protein expression of *EGFR* in SCLC compared to AC has been reported (Cerny et al. 1986; Sobol et al. 1987; Niederst et al. 2015; Shi et al. 2016). The heterogeneity of lung cancer tumors is well known and the small biopsies obtained may not always reflect the entire tumor entity. Therefore, a tumor is not seldom classified with the dominant histology where combinations of NSCLC and SCLC have been reported (Babakoohi et al. 2013; Lei et al. 2020; Vogelstein et al. 2013). Thus, it is possible that the plasma sample may illustrate a part of the tumor that was not detected in the tumor sample.

This study attempted to mimic the clinical real-world situation, increasing the clinical relevance of our findings. When analyzing cfDNA it is important to be able to evaluate the non-tumor variant background noise, which we addressed by the inclusion of a control group of patients that were investigated for lung cancer. To perform a more specific filtering of non-tumor variants matched white blood cells or machine learning algorithms would have been more preferable (Chabon et al. 2020). However, the cost of performing a paired deep sequencing on larger genomic regions is a considerable disadvantage for health economic reasons and even though machine learning has shown promise, it is still in an early phase.

The limitations of this study is in great part its size and the disadvantages of having clinical real-world data, which includes a sparse amount and quality of the obtained tissue or cytology. Therefore, further analyses using the same panel as with cfDNA was only possible for a small subset of patients and it is possible that several more variants in cfDNA could have been confirmed if more tumor sample had been available. It is evident that cfDNA has a clear clinical value even though some optimization is still needed in order to make confident calls about tumor origin of specific variants on a broad spectrum NGS in plasma.

## Conclusion

This study concludes that in the vast majority of advanced NSCLC patients a reliable variant analysis can be performed using liquid biopsy. The blood analysis provides additional molecular information to the tissue based analysis. This study also presents novel findings that the number of variants in plasma are associated with prognosis, possibly indicating a strategy for closer follow up on this crucial patient group. The data presented here could thereby aid effective personalized therapy for patients with lung cancer.

## Supplementary Information

**Additional file 1: Table S1.** Cohort characteristics and cfDNA MPS quality. Controls consisted of patients referred to the lung clinic with the suspicion of lung cancer but later diagnosed with benign lung diseases. UMD: Unique molecular depth; PS: ECOG Performance status; AC: Adenocarcinoma; SqCC: Squamous cell carcinoma; SCLC: Small cell lung cancer.

**Additional file 2.** Full VCF data.

## Data Availability

The cfDNA data can be found at the European Nucleotide Archive with Accession PRJEB45628. Variant Call Format files for all samples analyzed with NGS can be found in Additional file 2 attached to this article.

## Abbreviations

AF: Allele frequency
cfDNA: Circulating cell-free DNA
ctDNA: Circulating tumor DNA
CH: Clonal hematopoiesis
CNVs: Copy number variants
NGS: Next generation sequencing
TMB: Tumor mutational burden
UMD: Unique molecular depth
