# Supplementary material for: Liquid biopsy as an option for predictive testing and prognosis in patients with lung cancer
Source: Mol Med. 2021 Jul 3;27:68. doi: 10.1186/s10020-021-00331-1 (PMC8254966; doi:10.1186/s10020-021-00331-1)
Supplement: Supplementary file 1 — Additional file 1: Table S1. Cohort characteristics and cfDNA MPS quality. Controls consisted of patients referred to the lung clinic with the suspicion of lung cancer but later diagnosed with benign lung diseases. UMD: Unique molecular depth; PS: ECOG Performance status; AC: Adenocarcinoma; SqCC: Squamous cell carcinoma; SCLC: Small cell lung cancer. [file 10020_2021_331_MOESM1_ESM.docx]

**Table S1.** **Cohort characteristics and cfDNA MPS quality.** Controls consisted of patients referred to the lung clinic with the suspicion of lung cancer but later diagnosed with benign lung diseases. UMD: Unique molecular depth; PS: ECOG Performance status; AC: Adenocarcinoma; SqCC: Squamous cell carcinoma; SCLC: Small cell lung cancer.

| **Study ID** | **cfDNA input (ng)** | **cfDNA UMD** | **Diagnosis** | **Age** | **Sex** | **Smoking** | **Stage** | **Treatment** | **Follow up (months)** | **Survival status** | **PS** |
| --- | --- | --- | --- | --- | --- | --- | --- | --- | --- | --- | --- |
| 1 | 19.75 | 1298 | AC | 76 | Male | Never | II | Surgery | 27.84 | Diseased | 1 |
| 2 | 34.35 | 4623 | AC | 73 | Male | Current | IV | Chemotherapy | 7.67 | Diseased | 2 |
| 3 | 50.00 | 6605 | AC | 80 | Male | Current | I | Radiation | 7.41 | Diseased | 2 |
| 4 | 37.31 | 5307 | AC | 62 | Male | Former | IV | Chemotherapy | 46.66 | Sensored | 1 |
| 5 | 26.95 | 4744 | SqCC | 74 | Female | Former | IV | Chemotherapy | 4.56 | Diseased | 1 |
| 6 | 16.60 | 3611 | AC | 69 | Male | Former | II | Radiation | 42.00 | Sensored | 1 |
| 7 | 15.55 | 3548 | AC | 72 | Male | Current | I | Surgery | 33.18 | Diseased | 0 |
| 8 | 22.55 | 5244 | SCLC | 64 | Female | Current | IV | Radiation | 18.75 | Diseased | 0 |
| 9 | 11.90 | 2891 | AC | 47 | Female | Never | IV | Targeted therapy | 42.52 | Sensored | 0 |
| 10 | 50.00 | 7152 | SCLC | 72 | Male | Current | IV | Chemotherapy | 2.69 | Diseased | 2 |
| 11 | 50.00 | 7058 | SCLC | 80 | Female | Current | IV | Chemotherapy | 1.15 | Diseased | 2 |
| 12 | 11.50 | 2933 | SqCC | 58 | Male | Current | II | Surgery | 40.20 | Sensored | 0 |
| 13 | 12.70 | 3113 | SqCC | 70 | Male | Current | I | Surgery | 24.30 | Diseased | 1 |
| 14 | 50.00 | 6826 | AC | 81 | Male | Former | IV | Best supportive care/ no treatment | 1.61 | Diseased | 3 |
| 15 | 24.65 | 4277 | Control | 49 | Male | Never | NA | NA | NA | NA | NA |
| 16 | 37.45 | 6357 | SqCC | 73 | Male | Former | IIIa | Surgery | 16.69 | Diseased | 1 |
| 17 | 35.30 | 5771 | AC | 73 | Male | Never | IV | Best supportive care/ no treatment | 3.38 | Diseased | 3 |
| 18 | 50.00 | 7152 | AC | 51 | Female | Current | IV | Best supportive care/ no treatment | 0.75 | Diseased | 3 |
| 19 | 34.20 | 4457 | AC | 68 | Female | Never | IV | Chemotherapy | 37.11 | Sensored | 1 |
| 20 | 33.67 | 5602 | Control | 69 | Male | Former | NA | NA | NA | NA | NA |
| 21 | 22.70 | 6496 | AC | 61 | Male | Never | IV | Targeted therapy | 2.72 | Diseased | 2 |
| 22 | 22.70 | 4538 | SqCC | 73 | Female | Current | IIIa | Radiation | 33.70 | Sensored | 2 |
| 23 | 50.00 | 8011 | AC | 69 | Female | Former | IV | Best supportive care/ no treatment | 0.10 | Diseased | 4 |
| 24 | 20.95 | 4087 | AC | 74 | Female | Former | IIIa | Chemotherapy | 33.70 | Sensored | 1 |
| 25 | 50.00 | 6628 | AC | 57 | Female | Never | IV | Targeted therapy | 33.90 | Sensored | 0 |
| 26 | 50.00 | 7498 | Control | 83 | Male | Never | NA | NA | NA | NA | NA |
| 27 | 50.00 | 6273 | Control | 73 | Male | Current | NA | NA | NA | NA | NA |
| 28 | 46.85 | 5903 | SqCC | 71 | Female | Current | IV | Chemotherapy | 5.70 | Diseased | 1 |
| 29 | 44.65 | 7162 | AC | 68 | Female | Former | IV | Chemotherapy | 13.28 | Diseased | 1 |
| 30 | 20.10 | 1712 | Control | 84 | Male | Former | NA | NA | NA | NA | NA |
| 31 | 20.95 | 3306 | Control | 83 | Female | Never | NA | NA | NA | NA | NA |
| 32 | 25.80 | 4415 | AC | 69 | Male | Never | I | Surgery | 30.07 | Sensored | 1 |
| 33 | 29.05 | 5040 | AC | 62 | Female | Former | IV | Targeted therapy | 30.92 | Sensored | 1 |
| 34 | 9.30 | 2328 | SqCC | 75 | Male | Current | I | Surgery | 29.18 | Sensored | 0 |
| 35 | 20.00 | 4482 | AC | 57 | Male | Never | IIIb | Targeted therapy | 29.44 | Sensored | 0 |
| 36 | 50.00 | 5882 | AC | 79 | Male | Former | IV | Radiation | 1.25 | Diseased | 1 |
| 37 | 24.55 | 3337 | Control | 76 | Male | Current | NA | NA | NA | NA | NA |
| 38 | 50.00 | 6274 | AC | 81 | Male | Current | IV | Chemotherapy | 3.41 | Diseased | 2 |
| 39 | 40.50 | 6348 | SCLC | 71 | Male | Former | IV | Chemotherapy | 4.75 | Diseased | 2 |
| 40 | 24.70 | 4148 | Control | 81 | Female | Former | NA | NA | NA | NA | NA |
| 41 | 22.85 | 2614 | AC | 72 | Female | Never | IV | Targeted therapy | 28.20 | Sensored | 0 |
| 42 | 47.00 | 6877 | AC | 54 | Female | Former | IV | Chemotherapy | 5.02 | Diseased | 0 |
| 43 | 35.70 | 6565 | AC | 71 | Female | Current | I | Radiation | 23.25 | Sensored | 1 |
| 44 | 11.55 | 2972 | Control | 70 | Female | Former | NA | NA | NA | NA | NA |
| 45 | 20.90 | 3941 | Control | 71 | Female | Current | NA | NA | NA | NA | NA |
| 46 | 21.70 | 4365 | Control | 82 | Male | Never | NA | NA | NA | NA | NA |
| 47 | 24.80 | 4411 | Control | 76 | Male | Never | NA | NA | NA | NA | NA |
| 48 | 44.85 | 5949 | Control | 81 | Female | Former | NA | NA | NA | NA | NA |
| 49 | 31.40 | 6018 | Control | 71 | Male | Current | NA | NA | NA | NA | NA |
| 50 | 21.40 | 3600 | Control | 71 | Female | Never | NA | NA | NA | NA | NA |
| 51 | 33.40 | 4935 | AC | 69 | Female | Current | IV | Best supportive care/ no treatment | 2.46 | Diseased | 3 |
| 52 | 27.15 | 5518 | AC | 68 | Female | Former | IV | Immunotherapy | 24.82 | Sensored | 1 |
| 53 | 22.50 | 3982 | SqCC | 66 | Male | Former | IIIa | Radiation + Chemotherapy | 24.33 | Sensored | 0 |
| 54 | 15.05 | 3027 | SqCC | 75 | Male | Former | IIIa | Radiation + Chemotherapy | 9.54 | Diseased | 1 |
| 55 | 20.50 | 4095 | AC | 39 | Male | Never | IV | Targeted therapy | 24.16 | Sensored | 0 |
| 56 | 30.00 | 5758 | SqCC | 80 | Male | Former | I | Radiation | 22.43 | Sensored | 1 |
| 57 | 16.65 | 4633 | SqCC | 81 | Female | Former | IIIb | Radiation | 8.98 | Diseased | 2 |
| 58 | 18.75 | 3514 | SqCC | 58 | Male | Current | IV | Chemotherapy | 6.52 | Diseased | 0 |
| 59 | 31.30 | 4976 | AC | 68 | Male | Former | IIIb | Radiation | 20.20 | Diseased | 0 |
| 60 | 22.45 | 3490 | AC | 78 | Male | Former | I | Surgery | 22.46 | Sensored | 0 |
| 61 | 26.00 | 4750 | SqCC | 75 | Male | Former | IIIb | Radiation + Chemotherapy | 22.79 | Sensored | 2 |
| 62 | 24.65 | 4602 | SCLC | 72 | Female | Current | II | Radiation + Chemotherapy | 21.87 | Sensored | 1 |
| 63 | 43.95 | 6933 | SCLC | 66 | Female | Current | IIIb | Radiation + Chemotherapy | 12.36 | Diseased | 1 |
| 64 | 23.00 | 3626 | AC | 72 | Male | Former | IV | Chemotherapy | 8.30 | Diseased | 1 |
| 65 | 38.85 | 5917 | AC | 76 | Male | Former | IV | Chemotherapy | 15.21 | Sensored | 1 |
| 66 | 50.00 | 6654 | SCLC | 74 | Male | Former | IV | Best supportive care/ no treatment | 0.03 | Diseased | 3 |
| 67 | 50.00 | 6944 | AC | 85 | Female | Current | IV | Best supportive care/ no treatment | 1.77 | Diseased | 3 |
| 68 | 33.20 | 5624 | SqCC | 83 | Male | Former | IV | Best supportive care/ no treatment | 0.26 | Diseased | 3 |
| 69 | 25.55 | 9243 | AC | 73 | Male | Former | IV | Best supportive care/ no treatment | 0.13 | Diseased | 3 |
| 70 | 13.85 | 3139 | SqCC | 69 | Female | Former | IV | Immunotherapy | 4.03 | Diseased | 0 |
| 71 | 13.30 | 2566 | AC | 65 | Female | Never | I | Surgery | 7.97 | Sensored | 1 |
| 72 | 50.00 | 4676 | AC | 84 | Female | Never | IV | Targeted therapy | 7.11 | Sensored | 2 |
| 73 | 38.10 | 4724 | AC | 79 | Female | Former | II | Radiation + Chemotherapy | 3.90 | Sensored | 1 |
| 74 | 24.65 | 713 | Control | 52 | Female | Former | NA | NA | NA | NA | NA |
| 75 | 43.90 | 4563 | AC | 66 | Female | Former | IV | Chemotherapy | 2.69 | Sensored | 1 |
| 76 | 50.00 | 7665 | SCLC | 84 | Male | Former | IV | Chemotherapy | 2.33 | Sensored | 2 |
